# Ribosome Rescue Pathways in Bacteria

**DOI:** 10.3389/fmicb.2021.652980

**Published:** 2021-03-18

**Authors:** Claudia Müller, Caillan Crowe-McAuliffe, Daniel N. Wilson

**Affiliations:** Institute for Biochemistry and Molecular Biology, University of Hamburg, Hamburg, Germany

**Keywords:** ArfA, ArfB, ribosome rescue, ribosome-associated quality control, RqcH, tmRNA, SmpB, peptidyl-tRNA drop-off

## Abstract

Ribosomes that become stalled on truncated or damaged mRNAs during protein synthesis must be rescued for the cell to survive. Bacteria have evolved a diverse array of rescue pathways to remove the stalled ribosomes from the aberrant mRNA and return them to the free pool of actively translating ribosomes. In addition, some of these pathways target the damaged mRNA and the incomplete nascent polypeptide chain for degradation. This review highlights the recent developments in our mechanistic understanding of bacterial ribosomal rescue systems, including drop-off, *trans*-translation mediated by transfer-messenger RNA and small protein B, ribosome rescue by the alternative rescue factors ArfA and ArfB, as well as *Bacillus* ribosome rescue factor A, an additional rescue system found in some Gram-positive bacteria, such as *Bacillus subtilis*. Finally, we discuss the recent findings of ribosome-associated quality control in particular bacterial lineages mediated by RqcH and RqcP. The importance of rescue pathways for bacterial survival suggests they may represent novel targets for the development of new antimicrobial agents against multi-drug resistant pathogenic bacteria.

## Introduction

**FIGURE 1:**
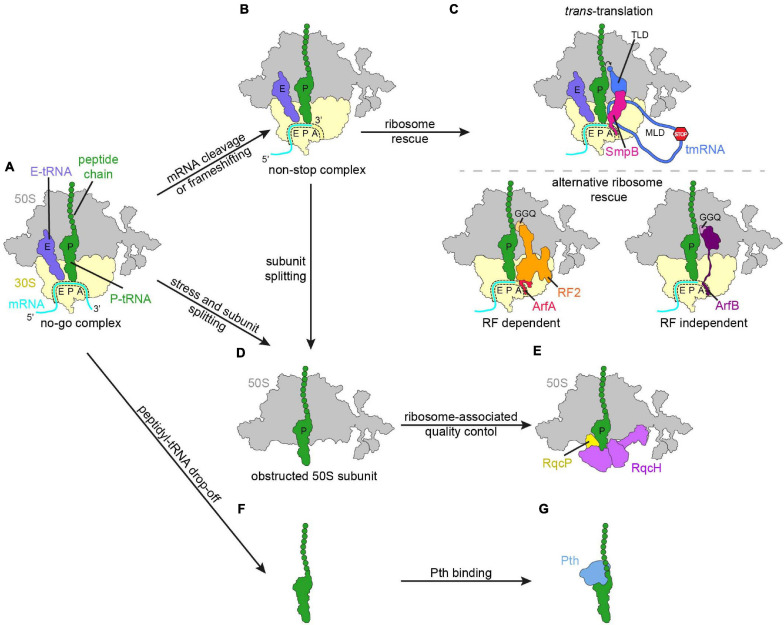
Schematic overview of regulation mechanisms in response to impeded or aberrant translation. **(A)** No-go ribosome complex (large subunit, 50S, gray; small subunit, 30S, yellow; mRNA, cyan; P-site tRNA with polypeptide chain, green; E-site tRNA, slate blue). Various responses to the translational pausing generating the no-go complex, like cleavage of the mRNA in the A-site or frameshifting can lead to the ribosome ending up in a non-stop complex **(B)**. **(C)** The non-stop complex is recognized by ribosome rescue mechanisms. **(D,E)** In the case that subunit splitting of the no-go complex or the non-stop complex occurred, the obstructed 50S subunit is subjected to bacterial ribosome-associated quality control. **(F)** Peptidyl-tRNA can drop-off from the ribosome and is recognized by peptidyl-tRNA hydrolase (Pth, blue) **(G)**.

When ribosomes encounter impediments during translation, they can stall on the mRNA instead of continuing protein synthesis. Environmental and cellular agents can cause impediments due to chemical mRNA damage for example by alkylation or oxidation (Wurtmann and Wolin, 2009; Simms and Zaher, 2016; Yan and Zaher, 2019; Thomas et al., 2020). Such impediments lead to the formation of so-called no-go complexes, in which the ribosome cannot proceed further on the mRNA (Figure 1A). It should be noted that stalling on intact mRNAs can also occur for instance due to rare codon stretches, translational misreading, problematic polypeptide stretches or weak termination codons (Hayes and Sauer, 2003; Sunohara et al., 2004; Li et al., 2006, 2007; Singh et al., 2008; Garza-Sánchez et al., 2009; Zaher and Green, 2009, 2010, 2011; Giudice and Gillet, 2013; Petropoulos et al., 2014; Sharma et al., 2014; Samatova et al., 2020). In many cases, these stalling events may be temporary and can be alleviated by the ribosome itself, or with help of specific translation factors (Zaher and Green, 2009, 2010, 2011; Doerfel et al., 2013; Peil et al., 2013; Ude et al., 2013; Petropoulos et al., 2014; Lassak et al., 2016; Huter et al., 2017a; Samatova et al., 2020), however, in some cases they result in prolonged stalling, which can therefore also be considered a form of no-go complex. In addition, ribosomes can also become stuck on mRNAs that lack a stop codon, so-called non-stop ribosomal complexes (Figure 1B). Ribosomes become stuck on non-stop mRNAs because they translate till the 3′ end of the mRNA but cannot continue elongating or enter the termination phase, since the mRNA ends in the P-site and there is no codon available in the A-site of the ribosome (Figures 1A,B) (Keiler et al., 1996). For example, readthrough of the stop codon (nonsense suppression), miscoding-inducing drugs or non-programmed frameshifting can lead to the lack of an in-frame stop codon, as well as spurious activity of RNases during mRNA turnover (Abo et al., 2002; Ueda et al., 2002; Bandyra and Luisi, 2013; Yan and Zaher, 2019; Thomas et al., 2020). Additionally, premature transcription termination can generate an incomplete or truncated mRNA that lacks a stop codon and is therefore also referred to as a non-stop mRNA. Since transcription and translation can occur simultaneously in bacteria (Wang et al., 2020; Washburn et al., 2020; and reviewed by Conn et al., 2019), any ribosomes translating the incomplete or truncated mRNA would also generate non-stop complexes. However, the processivity of RNA polymerase is high and these events are likely to be rare (Nudler et al., 1996). Moreover, no-go ribosomal complexes can be converted into non-stop complexes by mRNA cleavage in the vacant ribosomal A-site, which requires previous 3′ to 5′ mRNA degradation by RNase II or polynucleotide phosphorylase (Hayes and Sauer, 2003; Sunohara et al., 2004; Li et al., 2006, 2007; Garza-Sánchez et al., 2009). Cleavage of the mRNA in the A-site is also part of the bacterial stress response, where for instance, nutrient starvation induces expression of toxins, like the endonuclease RelE, which is a member of the RelBE toxin-antitoxin system (Christensen et al., 2001; Pedersen et al., 2003). Under normal conditions, the antitoxin RelB forms a complex that inactivates the toxin RelE; however, upon starvation RelB is degraded and the endonuclease RelE becomes active (Christensen et al., 2001). RelE then generates non-stop complexes by cleaving the mRNA in the A-site of the ribosome (Pedersen et al., 2003; Neubauer et al., 2009). Generation of non-stop complexes during starvation is likely to be a general mechanism to temporarily reduce the energy consumption of the cell, since protein production consumes ∼50% of energy in growing *Escherichia coli* cells (Russell and Cook, 1995; Pedersen et al., 2003; Garza-Sánchez et al., 2008). However, non-stop complexes need to be resolved when starvation is alleviated. Moreover, in *E. coli* growing in rich medium, ∼2–4% of peptidyl-tRNAs are not hydrolyzed, suggesting that no-go and non-stop events occur relatively frequently, even in the absence of stress (Ito et al., 2011).

Bacteria have evolved a diverse array of mechanisms to rescue no-go and non-stop ribosomal complexes (Figure 1). This is critical for survival in bacteria since otherwise ribosomes and tRNAs become sequestered from the pool of free translational components and the capacity of the cell to produce proteins rapidly diminishes (Keiler et al., 1996; Karzai et al., 1999; Tenson et al., 1999; Moore and Sauer, 2005; Chadani et al., 2010, 2011b; Goralski et al., 2018; Shimokawa-Chiba et al., 2019). The best characterized bacterial rescue systems that resolve no-go and non-stop ribosomes include *trans*-translation (Figure 1C, upper panel), alternative ribosome rescue factors (Arfs) (Figure 1C, lower panel), the recently identified bacterial ribosome quality control (RQC; Figures 1D,E) and peptidyl-tRNA drop-off (Figures 1F,G; Menninger, 1976; Keiler et al., 1996; Karzai et al., 1999; Chadani et al., 2010, 2011b; Handa et al., 2011; Goralski et al., 2018; Shimokawa-Chiba et al., 2019).

The major pathway for the rescue of non-stop ribosomal complexes is *trans*-translation (Figure 1C, upper panel), which was identified in >99% of bacterial genomes and is mediated by transfer-messenger RNA (tmRNA, formerly 10S, 10Sa RNA or *ssrA* RNA) in complex with small protein B (SmpB) (Tu et al., 1995; Keiler et al., 1996; Karzai et al., 1999; Hudson et al., 2014). tmRNA has two functional units, namely a tRNA-like domain (TLD) and an mRNA-like domain (MLD). In complex with SmpB, the TLD of tmRNA mimics the structure of tRNA^*Ala*^, which allows it to be charged with alanine, and to bind to the vacant ribosomal A-site of a non-stop complex (Komine et al., 1994; Ushida et al., 1994). The messenger part of tmRNA harbors a short reading frame encoding a degradation tag, followed by a stop codon (Keiler et al., 1996). After peptidyl transfer to the TLD, the ribosome resumes translation on the messenger-part, terminates on the stop codon and canonical ribosome recycling presumably then occurs (Keiler et al., 1996; Rae et al., 2019). The mRNA and the nascent peptide originating from the non-stop complex are targeted for degradation by means of *trans*-translation, so that all components of the non-stop complex are recycled or removed during the process (Keiler et al., 1996; Yamamoto et al., 2003). In some pathogenic bacteria, like *Neisseria gonorrhoeae* or *Mycobacterium tuberculosis*, *trans*-translation is essential, whereas others have evolved protein-based Arfs (Figure 1C, lower panel; Huang et al., 2000; Keiler and Feaga, 2014; Personne and Parish, 2014).

The Arf mechanisms can be divided in release factor (RF)-dependent and RF-independent mechanisms, as alternative ribosome rescue factor A (ArfA) recruits RF2 to hydrolyze the nascent polypeptide chain from the P-site tRNA, while ArfB can perform hydrolysis itself (Chadani et al., 2010, 2011b, 2012; Handa et al., 2011). In contrast to *trans*-translation, the non-stop mRNA and the polypeptide are not targeted for degradation by the Arf mechanisms, hence *trans*-translation is often considered as superior to Arfs and phenotypes can be observed upon deletion of tmRNA (*ssrA* gene) or SmpB (*smpB* gene) (Komine et al., 1994; Muto et al., 2000; Moore and Sauer, 2007; Keiler, 2008; Svetlanov et al., 2012; Keiler and Feaga, 2014). However, it has been shown that by changing the tag sequence of tmRNA to a degradation-deficient variant, it is the resolution of the non-stop complexes, rather than the degradation of the polypeptide, that ensures the survival of the cell (Huang et al., 2000; Chadani et al., 2010). The phylogenetic distribution of ArfA is restricted to a subset of β- and γ-proteobacteria, while ArfB has a wider distribution and is found in 34% of a set of representative bacterial genomes drawn from 21 phyla (Schaub et al., 2012; Feaga et al., 2014). In *Francisella tularensis*, *Staphylococcus aureus*, and *Bacillus subtilis trans*-translation is not essential, yet no apparent Arf homolog exists, leading to the speculation that this could hint at further alternative ribosome rescue systems (Shin and Price, 2007; Liu et al., 2010; Svetlanov et al., 2012; Keiler and Feaga, 2014). This was supported by recent studies, which identified ArfT in *F. tularensis* and *Bacillus* ribosome rescue factor A (BrfA) in *B. subtilis*, which are both RF-dependent alternative rescue systems (Goralski et al., 2018; Shimokawa-Chiba et al., 2019). Like ArfB, ArfT has a broader phylogenetic distribution (Burroughs and Aravind, 2019). Interestingly, ArfT cooperates with both RF1 and RF2 for hydrolysis of the nascent chain (Goralski et al., 2018). BrfA is likely limited to the *Bacillus* genus and exclusively recruits RF2, and hence BrfA has some similarities but also differences to ArfA (Shimokawa-Chiba et al., 2019). It is unclear whether an unidentified Arf system exists in *S. aureus*, however, *S. aureus* also appears to have the RQC pathway (discussed below; Lytvynenko et al., 2019).

The bacterial RQC pathway was discovered originally in *B. subtilis* (Figures 1D,E) and is partially redundant with *trans-*translation (Lytvynenko et al., 2019). Similar to eukaryotic RQC (reviewed by Joazeiro, 2019 and Yan and Zaher, 2019), bacterial RQC acts on large subunits bearing peptidyl-tRNAs, tagging and targeting the aberrant nascent peptide for degradation, thereby freeing and recycling the large subunit for the next round of translation (Lytvynenko et al., 2019; Crowe-McAuliffe et al., 2021; Filbeck et al., 2021). Bacterial RQC involves RqcH, a NEMF-family protein and homolog of the eukaryotic RQC factor Rqc2. NEMF family proteins are also found in archaea, although they have been lost in a few bacterial lineages, including *E. coli*. The near-universal phylogenetic distribution of the factor, as well as its conserved domain organization, structure and the functional similarity between prokaryotic RqcH and eukaryotic Rqc2/NEMF imply that the factor was already present in the last common universal ancestor (LUCA) (Burroughs and Aravind, 2014; Lytvynenko et al., 2019; Crowe-McAuliffe et al., 2021). At present, the relationship between RQC and other bacterial ribosome rescue systems remains unclear, mainly due to the lack of direct information as to which factor and/or conditions generate the peptidyl-tRNA-large subunit complexes. The redundancy with trans-translation suggests that bacterial RQC acts on some non-stop complexes. However, other stalled ribosomal complexes may also act as RQC substrates.

In cases where the ribosome stalls with a short oligopeptide nascent chain (<7 amino acids), the peptidyl-tRNA may dissociate from the ribosome without undergoing canonical translation termination (Figure 1F; Dinçbas et al., 1999; Heurgué-Hamard et al., 2000; Gonzalez de Valdivia and Isaksson, 2005). For example, *bar* mini-genes encoded by the bacteriophage λ contain a start codon, followed by AUA and then immediately UAA, thereby encoding the dipeptide formylmethionine-isoleucine (Ontiveros et al., 1997; Hernández-Sánchez et al., 1998). Expression of *bar* is toxic to *E. coli* cells deficient in peptidyl-tRNA hydrolase (Pth), and can cause accumulation of peptidyl-tRNA, which reduces the cellular pool of free tRNA for aminoacylation (Tenson et al., 1999). Additionally, some upstream leader peptides, macrolide antibiotics, as well as certain patterns of codons soon after the start codon, can trigger peptidyl-tRNA drop-off (Gonzalez de Valdivia and Isaksson, 2005; Gong et al., 2007; Jacinto-Loeza et al., 2008). Drop-off may be spontaneous or promoted by various translation factors, among them the ribosome recycling factor (RRF), elongation factor G (EF-G) and release factor 3 (RF3) (Heurgué-Hamard et al., 1998; Rao and Varshney, 2001; Gong et al., 2007; Singh et al., 2008; Watanabe et al., 2010; Vivanco-Domínguez et al., 2012). After peptidyl-tRNA drop-off from the ribosome the ester bond between the nascent polypeptide chain and the tRNA is targeted by Pth (Figure 1G), which prevents the cell from toxic effects of peptidyl-tRNA accumulation (Cuzin et al., 1967; Kossel and RajBhandary, 1968; Atherly, 1978; Menninger, 1979; Menez et al., 2000; Vivanco-Domínguez et al., 2006). Pth is essential in *E. coli* and Pth enzymes can be found in all domains of life, emphasizing the importance of avoiding peptidyl-tRNA accumulation (Kossel and RajBhandary, 1968; Jost and Bock, 1969; Atherly and Menninger, 1972; Menninger et al., 1974; Keiler et al., 1996; Rosas-Sandoval et al., 2002; Hayes and Sauer, 2003; Singh and Varshney, 2004; Sunohara et al., 2004; Baba et al., 2006). In the following sections ribosome rescue mechanisms and bacterial RQC will be described in greater detail. Due to the relatively little knowledge about ArfT, this mechanism will not be elucidated further.

## Bacterial Ribosome Rescue Systems

### Trans-Translation

**FIGURE 2:**
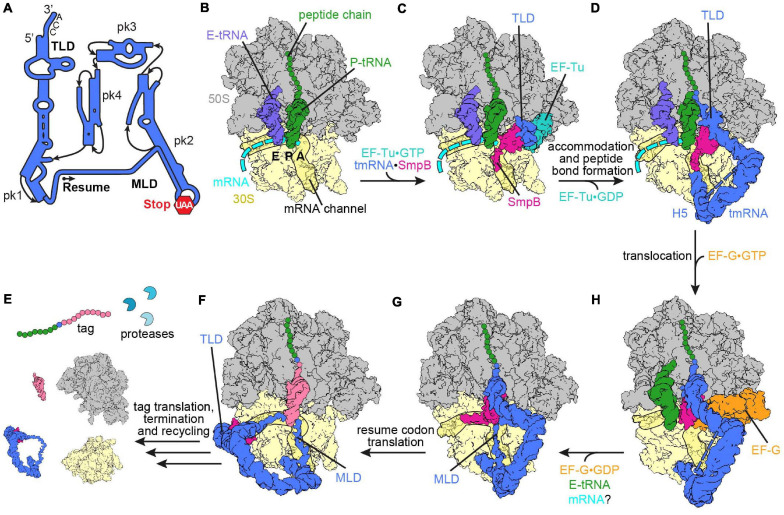
Ribosome rescue by *trans*-translation. **(A)** Schematic representation of tmRNA (pk, pseudoknot; TLD, tRNA-like domain; MLD, mRNA-like domain). The arrow marks the resume codon and the red hexagon indicates the stop codon (UAA) of the MLD. **(B)** Non-stop complex (large subunit, 50S, gray; small subunit, 30S, yellow) with peptidyl-tRNA (green) in the P-site, E-site tRNA (slate blue) and mRNA (cyan) [PDB ID 4V8Q (Neubauer et al., 2012)]. **(C)** Delivery of the tmRNA-TLD (blue) in complex with SmpB (violet red) to the non-stop complex by EF-Tu (light sea green) [PDB ID 4V8Q (Neubauer et al., 2012)]. **(D)** tmRNA⋅SmpB (blue and violet red, respectively) accommodated to the A-site of a non-stop complex [PDB ID 6Q97 (Rae et al., 2019)]. Helix 5 (H5) of tmRNA binds close to the mRNA entry channel. The flexible MLD is indicated by the dashed line. **(E)** Post-translocation intermediate state of tmRNA⋅SmpB with EF-G (orange) [PDB ID 4V6T (Ramrath et al., 2012)]. tmRNA⋅SmpB and the tRNA (green) are in ap/P and pe/E hybrid states, respectively. **(F)** Post-translocation complex with tmRNA⋅SmpB in the P-site [PDB ID 6Q98 (Rae et al., 2019)]. The C-terminal tail of SmpB occupies the E-site of the mRNA channel and the resume codon is placed in the A-site. **(G)** Translation of the resume codon has occurred and the peptide chain was transferred from the TLD to the tRNA (pale violet red) decoding the resume codon [PDB ID 6Q9A (Rae et al., 2019)]. The TLD and SmpB are past the E-site at the outside of the ribosome, while the MLD is fully loaded to the mRNA channel. **(H)** Translation of the tag peptide, termination at the MLD stop codon and subsequent ribosome recycling have occurred. The tagged peptide is targeted by proteases.

***Trans*-translation** is the major pathway of ribosome rescue in bacteria and is mediated by the ribonucleoprotein complex of tmRNA and SmpB. tmRNA was already discovered in 1979 as one of the stable RNAs present in *E. coli* (Ray and Apirion, 1979), but its function and the cooperation with SmpB was described later in the 1990s (Tu et al., 1995; Keiler et al., 1996; Karzai et al., 1999). Subsequently, tmRNA was named after its properties, as it contains both a tRNA-like and the MLD connected by several pseudoknots (Figure 2A; Atkins and Gesteland, 1996; Felden et al., 1996). Usually, tmRNA is a single RNA molecule of approximately 360 nucleotides, but in some bacterial species tmRNA is composed of two RNA chains (Williams and Bartel, 1996; Keiler et al., 2000). However, the secondary structure and the function are conserved throughout bacteria. In single-molecule tmRNAs the TLD consists of the 5′ and 3′ ends, which fold into a secondary structure that resembles tRNA^*Ala*^ without the anticodon stem loop (ASL) (Komine et al., 1994; Ushida et al., 1994; Gutmann et al., 2003; Ramrath et al., 2012). This allows recognition and charging of the CCA-3′ end of the TLD with alanine by the canonical alanyl-tRNA synthetase (Komine et al., 1994). Moreover, the binding affinity of EF-Tu for the TLD and tRNAs is comparable (Rudinger-Thirion et al., 1999; Barends et al., 2000). tmRNA binds with high affinity to SmpB, which occupies the space of the missing ASL and, additionally, SmpB is required for *trans*-translation (Karzai et al., 1999; Gutmann et al., 2003; Cheng et al., 2010; Weis et al., 2010a; Neubauer et al., 2012). SmpB is a protein of ∼160 amino acids with a globular N-terminal domain (NTD) and a C-terminal tail that is unstructured in solution (Dong et al., 2002). The formation of the tmRNA⋅SmpB complex stabilizes the secondary structure of tmRNA and promotes the interaction with alanyl-tRNA synthetase (Karzai et al., 1999; Wiegert and Schumann, 2001; Keiler and Shapiro, 2003). The tmRNA MLD harbors the “tag reading frame,” which encodes a short degradation tag (“tag peptide”) that is translated by the ribosome to form the C-terminus of the nascent protein and varies in length (8–35 amino acids) in different species (Komine et al., 1994; Tu et al., 1995; Felden et al., 1996; Williams and Bartel, 1996; Keiler et al., 2000). The first alanine of the tag peptide (**A**ANDENYALAA in *E. coli*) is not encoded in the tag reading frame, but is rather the alanine attached to the TLD of tmRNA (Tu et al., 1995; Keiler et al., 1996). The tag reading frame differs from an ORF, since it does not include a start codon and instead begins with the so-called resume codon (GCA for alanine in *E. coli*). However, like an ORF, it ends with a canonical stop codon (UAA in *E. coli*) (Williams et al., 1999).

Biochemical and structural data acquired over the last three decades have painted a detailed picture of the mechanism of *trans*-translation. The ribonucleoprotein complex of tmRNA⋅SmpB is delivered to the A-site of the ribosome in a quaternary complex with EF-Tu⋅GTP (Figures 2B,C; Rudinger-Thirion et al., 1999; Barends et al., 2000; Valle et al., 2003; Fu et al., 2010; Weis et al., 2010b; Neubauer et al., 2012). Upon binding, the C-terminal tail of SmpB probes the mRNA entry channel (Neubauer et al., 2012), which is consistent with *in vitro* assays showing that the C-terminal tail is significant for events following delivery of tmRNA⋅SmpB to the non-stop complex by EF-Tu (Jacob et al., 2005; Sundermeier et al., 2005; Kurita et al., 2010; Miller et al., 2011). More precisely, the studies observed that the peptidyl transfer to the TLD of tmRNA was inhibited upon truncation or deletion of the C-terminal tail, which indicates that the tail supports accommodation of tmRNA⋅SmpB into the A-site. Additionally, it was observed that the rate of peptidyl transfer to the TLD of tmRNA was notably reduced with mRNAs extending more than 9 nucleotides past the P-site, in agreement with the rejection of tmRNA⋅SmpB from these complexes being increased (Ivanova et al., 2004; Asano et al., 2005; Kurita et al., 2014b). Furthermore, comparison of the path of a full-length mRNA and SmpB indicate a severe clash between both (Neubauer et al., 2012; Huter et al., 2017c). Taken together, the findings support that the preferred substrate for *trans*-translation is a non-stop complex with an empty mRNA entry channel as well as complexes with up to nine nucleotides following the P-site, but complexes with longer mRNAs are only targeted occasionally. The C-terminal tail of SmpB attaches to the surrounding 16S rRNA of the mRNA entry channel *via* positively charged residues and engages an α-helical conformation on the ribosome. One conserved stretch is the DKR-motif (Asp_137_Lys_138_Arg_139_ in *E. coli*). Although single mutations in this motif have only a marginal effect, the substitution of all three residues to alanine eliminated *trans*-translation activity *in vitro* and *in vivo* (Sundermeier et al., 2005; Miller et al., 2011). Similar to the aforementioned truncation and deletion of the C-terminal tail, the association with the ribosome was not abolished, but the tagging activity was strongly inhibited. The globular domain and the upper part of the C-terminal tail of SmpB also uses positively charged residues to interact with the decoding center of the ribosome (Neubauer et al., 2012). In addition, conserved aromatic residues of SmpB stack on the decoding bases G530 and A1493, whereas A1492 remains within helix 44 (h44) of the 16S rRNA where it stacks with the 23S rRNA nucleotide A1913 of helix 69 (H69) (*E. coli* numbering is used throughout the review). Mutations of the decoding bases (G530A, A1492G, or A1493G) were found to reduce the activity of *trans*-translation only twofold, whereas accommodation of aminoacyl-tRNA at the PTC was reduced ∼1000-fold (Miller et al., 2011). Hence, the binding of tmRNA⋅SmpB *via* stacking interactions is less dependent on the identity of these bases than is the stabilization and monitoring of the codon:anticodon interaction by H-bonds during decoding (Ogle et al., 2001, 2002; Neubauer et al., 2012). Overall, the conformation of the TLD and SmpB in the tmRNA⋅SmpB⋅EF-Tu⋅GTP quaternary complex in the A-site (Figure 2C) is similar to the A/T-state of aminoacyl-tRNA⋅EF-Tu⋅GTP during decoding (Schmeing et al., 2009; Neubauer et al., 2012). The pseudoknots of tmRNA were found to be wrapped around the head of the small subunit (Valle et al., 2003; Fu et al., 2010; Neubauer et al., 2012). Thereby, pseudoknot 2 and helix 5 of tmRNA are bound close to the entrance of the mRNA channel (Fu et al., 2010). These interactions remain throughout *trans*-translation and act as a flexible hinge and anchor tmRNA to the ribosome, whereas the rest of the pseudoknots change their position during the process (Rae et al., 2019; Figures 2D–F). In contrast to the release of the CCA-3′ end of aminoacyl-tRNAs from EF-Tu during elongation, kinetic studies indicate that the process for tmRNA⋅SmpB is less dependent on GTP hydrolysis (Kurita et al., 2014b; Miller and Buskirk, 2014), although EF-Tu binds to the GTPase activating center (GAC) located on the large subunit in both cases (Schmeing et al., 2009; Neubauer et al., 2012). The release of the TLD of tmRNA from EF-Tu and the role of GTP hydrolysis in this regard need to be further evaluated in the future.

After accommodation of the TLD at the A-site of the PTC, transpeptidation occurs and the nascent polypeptide chain is transferred from the P-site tRNA to the alanine on the TLD of tmRNA⋅SmpB (Figure 2D; Tu et al., 1995; Keiler et al., 1996; Fu et al., 2010; Weis et al., 2010a; Ramrath et al., 2012). As during canonical translation elongation, translocation of tmRNA⋅SmpB is assisted by EF-G. A cryo-EM study revealed how domain IV of EF-G binds adjacent to the globular domain of SmpB (Figure 2E; Ramrath et al., 2012). As in translocation of A- and P-site tRNAs, the tmRNA⋅SmpB complex and the P-site tRNA also adopt ap/P and pe/E chimeric hybrid states, respectively. During translocation, the MLD has to be loaded into the mRNA channel, and therefore must pass through the A-site (or 30S) latch between h34 of the head and the G530-loop of the body (Ramrath et al., 2012; Rae et al., 2019). Ramrath et al. (2012) suggested that opening of the latch occurs due to a “unique extra-large swivel of the 30S head,” which encompasses an additional incline of the head in addition to the head swivel observed during canonical translocation. In the POST-translocation ribosome, the TLD and SmpB were located in the P-site, with the C-terminal helix of SmpB extending toward the E-site (Figure 2F; Rae et al., 2019). The resume codon of the MLD was placed in the A-site. Cryo-EM and mutational studies have indicated that the first five bases upstream of the MLD interact with SmpB, which is important for proper placement of the resume codon (Lee et al., 2001; Konno et al., 2007; Rae et al., 2019). Subsequently, a cognate tRNA binds to the resume codon, leading to peptide bond formation and translocation (Figure 2G). Ramakrishnan and co-workers revealed that the TLD and SmpB move beyond the E-site after the second translocation step. This appears to be necessary since the TLD-SmpB complex would clash with the ribosome in the E-site, hence tmRNA⋅SmpB does not mimic a tRNA in the E-site in the POST-translocation state (Rae et al., 2019). Instead, the MLD is fully loaded into the mRNA channel by passing through a second latch (E-site latch) between the ribosomal protein uS7 of the small subunit head, as well as uS11 and the 16S rRNA nucleotide G693 of the body (Figure 2G). Whether or not an “extra-large swivel” of the head domain during the second translocation accompanies the mRNA loading is not clear yet, but it would be a possible way to open the E-site latch.

Subsequently, translation continues on the MLD, adding the tag peptide to the nascent polypeptide chain, until the stop codon at the end of the tag reading frame enters the A-site (Tu et al., 1995; Keiler et al., 1996). Termination and ribosome recycling then ensue (Figure 2H). The tag peptide added to the polypeptide by *trans*-translation is recognized by several proteases, like ClpXP, ClpAP, and Lon, which promote rapid degradation of the aberrant nascent peptide (Gottesman et al., 1998; Flynn et al., 2001; Choy et al., 2007). Additionally, the defective mRNA is targeted for degradation by ribonuclease R (RNase R), which is required for mRNA decay and is enriched in non-stop complexes (Mehta et al., 2006; Richards et al., 2006; Ge et al., 2010; Venkataraman et al., 2014a, b). Recruitment of RNase R is mediated by the 3′ end of the tmRNA MLD. The mechanism of the recruitment is not yet clear, however mutations in the 3′ end region of the tag reading frame prohibit the targeting of RNase R (Venkataraman et al., 2014b). Furthermore, the mechanism of handing over the mRNA from the non-stop complex to RNase R still needs to be elucidated. According to Rae et al. (2019), the mRNA is already ejected during the first translocation step. RNase R is a 3′–5′ exonuclease and the 3′-end of the mRNA remains inside the ribosome and thus is protected until its ejection. The recruitment of RNase R to the pre-translocation non-stop complex would bring RNase R within close proximity to the target mRNA prior to ejection, which could be one way to promote degradation (Keiler, 2015). The degradation of the polypeptide and the mRNA is an advantage of tmRNA over the alternative ribosome rescue mechanisms (Hudson et al., 2014). Resolving the non-stop complexes by alternative ribosome rescue mechanisms ensures the survival of the bacterial cell in absence of *trans-*translation (Feaga et al., 2014; Keiler and Feaga, 2014), but leads to different phenotypes, particularly under stress conditions, depending on the species, for example, deletion of *trans*-translation in *E. coli* leads to increased sensitivity to antibiotic stress, whereas *B. subtilis* is rendered temperature sensitive, and *F. tularensis* displayed virulence defects (Abo et al., 2002; Shin and Price, 2007; Svetlanov et al., 2012; Li et al., 2013).

### Alternative Ribosome Rescue Factors

#### Release Factor-Dependent Alternative Ribosome Rescue Factors

**Alternative ribosome rescue factor A (ArfA, formerly YhdL)** was discovered in a synthetic lethality screen performed in *E. coli* where the *ssrA* gene, which encodes tmRNA, was deleted (Chadani et al., 2010). This study showed that ArfA is essential in the absence of tmRNA, i.e., *trans*-translation, and *vice versa*. ArfA expression was found to be regulated by *trans*-translation (Garza-Sánchez et al., 2011; Schaub et al., 2012). The *arfA* gene encodes 72 amino acids, but contains a stem loop structure, which can cause premature transcription termination or serves as a target for specific cleavage by RNase III, leading to a non-stop mRNA in both cases (Chadani et al., 2011a; Garza-Sánchez et al., 2011; Schaub et al., 2012). Even if full-length *arfA* mRNA and ArfA protein is produced occasionally, the C-terminal amino acids are highly hydrophobic, rendering full-length ArfA aggregation prone and instable, with a short half-life of 1.6 min (Chiti, 2006; Chadani et al., 2011a). However, the majority of ArfA translation originates from truncated mRNA, which ends up in a non-stop complex and is subjected to *trans*-translation (Chadani et al., 2011a; Garza-Sánchez et al., 2011; Schaub et al., 2012). The C-terminally truncated ArfA protein escapes the ribosome only if *trans*-translation is defective or overwhelmed. The truncated ArfA (referred to as ArfA afterward) usually lacks the last 17–18 amino acids encoded by its ORF but nonetheless maintains full activity (Chadani et al., 2010, 2011a, b; Garza-Sánchez et al., 2011; Schaub et al., 2012). This indicates that the terminal amino acids are mainly important for regulation and not for activity, which is in line with their poor conservation (Chadani et al., 2011a; Garza-Sánchez et al., 2011; Schaub et al., 2012). The production of ArfA from truncated mRNA and thus the regulation by *trans*-translation indicates that it is indeed a back-up mechanism for *trans*-translation.

In a cell extract-based translation assay, ArfA was able to release a truncated peptide produced by a model non-stop mRNA, while a mutation of alanine 18 to threonine (A18T) in ArfA abolished the release activity and caused the synthetic lethal phenotype (Chadani et al., 2010). In follow-up studies, it was shown that ArfA could not release the peptide itself, and the release activity was specifically dependent on the presence of RF2 (Chadani et al., 2012; Shimizu, 2012; Kurita et al., 2020). During canonical termination, RF2 initially binds to the ribosome in a closed conformation, while the serine-proline-phenylalanine (SPF)-motif of the decoding loop in domain II recognizes the stop codon in the A-site (Svidritskiy and Korostelev, 2018; Fu et al., 2019). Afterwards, the conformation of RF2 changes to the so-called open conformation, which guides domain III into the PTC (Korostelev et al., 2008; Svidritskiy and Korostelev, 2018; Fu et al., 2019). Domain III harbors a glycine-glycine-glutamine (GGQ)-motif that is necessary for the hydrolysis of the polypeptide chain from the P-site tRNA (Korostelev et al., 2008; Weixlbaumer et al., 2008; Santos et al., 2013). ArfA, but also ArfA(A18T), are able to bind to non-stop complexes and to recruit RF2 but ArfA(A18T) failed to activate the hydrolysis activity of RF2 (Chadani et al., 2010, 2011b; Shimizu, 2012). Interestingly, the release activity of ArfA/RF2 was independent of the SPF-motif of RF2, suggesting that ArfA does not mimic a stop codon. In contrast, the GGQ-motif was essential as in canonical termination (Chadani et al., 2012). ArfA was found to be associated with isolated large subunits, which could reflect an initial binding site even in the absence of the small subunit (Chadani et al., 2010; Kurita et al., 2014a), but may not be physiologically relevant. Indeed, ArfA binding was mapped only to the small subunit by hydroxyl radical probing using a non-stop complex as substrate (Kurita et al., 2014a). Moreover, the pattern of the hydroxyl radical probing map differed in the presence of RF2. In particular, the pattern of the ArfA C-terminus remained unchanged and the binding was mapped to the mRNA entry channel in an overlapping position to SmpB and the mRNA path in the channel, indicating that ArfA monitors the mRNA entry channel as well. By contrast, the N-terminal interactions around the decoding center altered when RF2 was bound and suggested the induction of a stable productive conformation for both ArfA and RF2.

**FIGURE 3:**
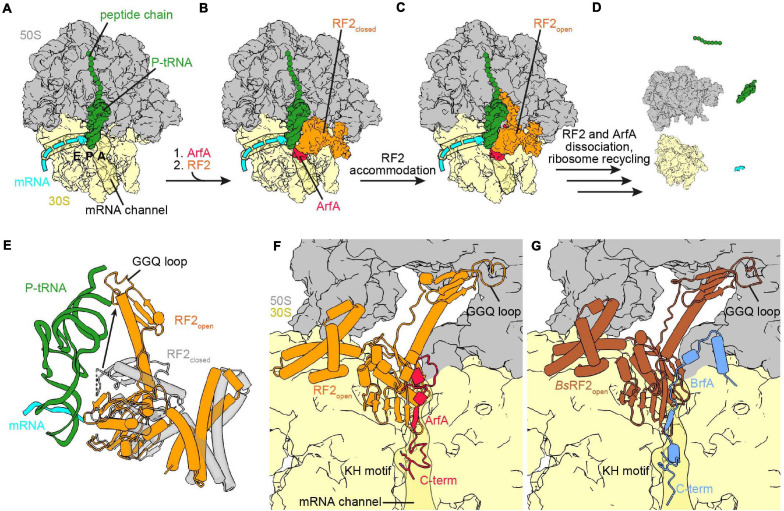
Ribosome rescue by ArfA and BrfA. **(A)** Non-stop complex (large subunit, 50S, gray; small subunit, 30S, yellow) with peptide chain and P-site tRNA (both green) and mRNA (cyan) [PDB ID 5MDW (James et al., 2016)]. **(B)** ArfA (red) binds first to the vacant ribosomal A-site and the mRNA entry channel and recruits RF2 (orange) in the closed conformation to the ribosome [PDB ID 5MDW (James et al., 2016)]. **(C)** ArfA and RF2 on the non-stop complex after the transition of RF2 to the open conformation [PDB ID 5MGP (Huter et al., 2017c)]. **(D)** ArfA and RF2 dissociated after release of the peptide chain and ribosome recycling has occurred. **(E)** Comparison of the RF2 closed [transparent gray, PDB ID 5MDW (James et al., 2016)] and open [orange, PDB ID 5MGP (Huter et al., 2017c)] conformation with P-site tRNA and mRNA for reference. **(F,G)** View into the A-site with bound ArfA and RF2 [PDB ID 5MGP (Huter et al., 2017c)] **(F)** or BrfA (blue) and *B. subtilis* RF2 [brown, PDB ID 6SZS (Shimokawa-Chiba et al., 2019)] **(G)**. Both RF2 are in the open conformation and the C-termini of ArfA and BrfA extend into the mRNA channel, the KH motif and the GGQ-loop are indicated.

Five cryo-EM studies provided detailed insight into the rescue mechanism by ArfA and RF2 recruitment to non-stop complexes (James et al., 2016; Demo et al., 2017; Huter et al., 2017c; Ma et al., 2017; Zeng et al., 2017). Collectively, the results indicated that ArfA recruits RF2 initially in a closed conformation (Figures 3A,B) and subsequently stabilizes an active open conformation (Figure 3C), which enables hydrolysis of the polypeptide chain from the P-site tRNA. Presumably, following hydrolysis, ArfA and RF2 dissociate and the ribosome is recycled by RRF and EF-G analogously to canonical post-termination complexes (Figure 3D). In all studies, C-terminally truncated ArfA was used, which was shortened by 12 (James et al., 2016; Demo et al., 2017) or 17 amino acids (Huter et al., 2017c; Ma et al., 2017; Zeng et al., 2017). Despite the different ArfA length of 55–60 residues, the density only allowed modeling of 46–48 amino acids due to flexibility of the C-terminus. This suggests that these residues are less important for association with the ribosome and is consistent with their poor conservation (Chadani et al., 2011a; Garza-Sánchez et al., 2011; Schaub et al., 2012). The position of the C-terminus within the mRNA entry channel overlaps the path of a full-length mRNA, providing further support for the hypothesis that the C-terminus of ArfA monitors the mRNA entry channel (Kurita et al., 2014a; James et al., 2016; Demo et al., 2017; Huter et al., 2017c; Ma et al., 2017; Zeng et al., 2017). In contrast to the defined path of the α-helical tail of SmpB (Neubauer et al., 2012) the C-terminus of ArfA forms a loop that obstructs the mRNA entry channel (James et al., 2016; Demo et al., 2017; Huter et al., 2017c; Ma et al., 2017; Zeng et al., 2017). This could explain the difference between *trans*-translation and ArfA in the ability to act on non-stop complexes with extension of the mRNA past the P-site. While *trans*-translation was shown to act with reduced activity on complexes with more than nine nucleotides downstream of the P-site, the activity of ArfA is already strongly reduced by more than three nucleotides past the P site (Ivanova et al., 2004; Asano et al., 2005; Shimizu, 2012; Kurita et al., 2014b; Zeng and Jin, 2016). This is in agreement with the structural data showing that the ArfA C-terminus allows maximal three nucleotides in the decoding center (James et al., 2016; Demo et al., 2017; Huter et al., 2017c; Ma et al., 2017; Zeng et al., 2017). Similar to SmpB, ArfA binds to the mRNA entry channel *via* hydrogen-bonding of positively charged amino acids with the surrounding 16S rRNA in a redundant manner, so that single mutations do not interfere with binding (Sundermeier et al., 2005; Miller et al., 2011; Kurita et al., 2014a; Ma et al., 2017; Zeng et al., 2017). By contrast, shortening ArfA by 32 C-terminal amino acids, leaving only 40 amino acids of ArfA, abolished rescue activity (Chadani et al., 2011a), indicating that removing positively charged stretches cannot be compensated for and reduces binding to the mRNA entry channel.

James et al. (2016) and Demo et al. (2017) were able to observe the recruitment of RF2 in the closed conformation (Figure 3E) using different approaches. While for Korostelev and co-workers the closed conformation appeared as a subset of their dataset (Demo et al., 2017), Ramakrishnan and co-workers formed two additional complexes with ArfA(A18T) and *Thermus thermophilus* RF2 (James et al., 2016), respectively. Comparison of the closed conformation of RF2 upon stop-codon recognition or recruitment by ArfA based on the alignment of the 16S rRNA showed that RF2 is in the same overall conformation in either case (James et al., 2016; Demo et al., 2017; Fu et al., 2019). The ArfA residues glutamine 27 (Q_27_) to glutamic acid 30 (E_30_) mediate the recruitment of RF2 by augmenting a β-strand to the β-sheet formed by the superdomain II/IV of RF2. Additionally, phenylalanine 25 (F_25_) of ArfA implements further hydrophobic interactions with the β-strands β4 and β5 within the superdomain II/IV of RF2 (James et al., 2016; Demo et al., 2017). The latter hydrophobic interactions are not conserved in RF1, providing an explanation for the specific binding of RF2 by ArfA (Shimizu, 2012; James et al., 2016; Huter et al., 2017c). This hypothesis was supported to some extent by a recent mutational study determining residues involved ArfA mediated RF2 recruitment and activation (Kurita et al., 2020). The upper middle part of ArfA (residue 15–26) contains a short α-helix (residue 15–24) and establishes initial interactions with the 16S rRNA at the decoding center. The N-terminus of ArfA (amino acids 1–14), as well as the switch loop and the GGQ-loop of RF2 are flexible when RF2 is closed (James et al., 2016; Demo et al., 2017). In contrast, the switch loop, the GGQ-loop and the N-terminus of ArfA are ordered when RF2 is in the open conformation (Figure 3F; James et al., 2016; Demo et al., 2017; Huter et al., 2017c; Ma et al., 2017; Zeng et al., 2017). Comparison of the complexes formed with ArfA and ArfA(A18T) showed that ArfA(A18T) failed to activate RF2 because the threonine mutation caused a steric hindrance, which prevented the ordering of the ArfA(A18T) N-terminus, as well as the associated rearrangement of the RF2 switch loop (James et al., 2016). As for canonical termination, this supports a role for the switch loop in the closed to open transition (James et al., 2016; Demo et al., 2017; Fu et al., 2019). Overall, the open conformation in the presence of ArfA resembles the open conformation during termination, with domain III of RF2 reaching toward the PTC and accommodating the GGQ motif for hydrolysis (James et al., 2016; Demo et al., 2017; Huter et al., 2017c; Ma et al., 2017; Zeng et al., 2017). One difference is a shift of the decoding loop due to the ArfA β-strand addition to the superdomain II/IV of RF2 (Huter et al., 2017c). ArfA does not interact with the SPF-motif, which is consistent with biochemical data indicating that mutation of the motif does not influence rescue by ArfA (James et al., 2016; Demo et al., 2017; Huter et al., 2017c; Zeng et al., 2017). Thus, ArfA does not mimic a stop codon in order to initiate the open conformation of RF2, but rather ArfA provides the platform for the conformational change itself. In comparison to the closed conformation, ArfA and open RF2 pack more tightly against the decoding center, which adopts a similar conformation as seen with SmpB, but is different from the conformation during stop codon recognition (James et al., 2016; Demo et al., 2017; Huter et al., 2017c). As in termination, helix α7 of RF2 is extended by an unstructured to structured transition of the switch loop, but ArfA promotes formation of a longer helical segment. Interestingly, it was recently found that RF2 retains activity in ribosome rescue and termination when the switch loop was swapped to the switch loop of RF1 (Kurita et al., 2020). However, exchanging the switch loop of RF1 with the one from RF2 does not enable RF1 to be activated by ArfA indicating that additional determinants are necessary to take part in ribosome rescue. Indeed, certain domain combinations allow RF1 recruitment and activation by ArfA (Kurita et al., 2020). Analogous to termination, placement of the GGQ motif into the PTC induces exposure of the ester bond between the P-site tRNA and the peptide for hydrolysis (James et al., 2016; Demo et al., 2017; Huter et al., 2017c; Ma et al., 2017; Zeng et al., 2017). Furthermore, methylation of Q_252_ of the GGQ motif enhanced peptide release during termination and ribosome rescue by ArfA/RF2, promoting a distinct conformation of Q_252_ in both cases (Zeng and Jin, 2016, 2018). In contrast to termination, the dissociation of RF2 was not accelerated by RF3 during ArfA mediated rescue (Zeng and Jin, 2016). The reasons for this difference, as well as the dissociation mechanism are still unknown and might be the subject of future studies.

***Bacillus* ribosome rescue factor A (BrfA, previously YqkK)** was recently identified and characterized as a ribosome rescue factor by Shimokawa-Chiba et al. (2019). The involvement of BrfA in ribosome rescue was found by a synthetic lethality screen in *B. subtilis* deficient in *trans-*translation. The *brfA* gene encodes 72 amino acids, but contains a rho-independent transcription terminator before the stop codon. Using a reporter construct with a *lacZ* gene downstream of *brfA* variants it was shown that transcription termination and subsequent degradation by *trans-*translation occurred using the wild-type construct. Additionally, disruption of the rho-independent transcription terminator by synonymous mutation, as well as deletion of the terminator region led to LacZ expression. Hence, BrfA is likely expressed from a truncated mRNA and regulated by *trans-*translation in a similar fashion to ArfA, so that the active BrfA protein consists of ∼62 amino acids. Furthermore, BrfA was shown to cooperate with RF2 in the course of rescue of non-stop complexes *in vitro* (Shimokawa-Chiba et al., 2019). Similar to ArfA, the release activity was dependent on the GGQ motif of RF2, whereas the SPF-motif of the decoding loop was dispensable. This indicates that BrfA does not mimic a stop codon and therefore hydrolysis activity of RF2 is enabled in an alternative fashion. BrfA can support peptidyl-tRNA hydrolysis by *B. subtilis* RF2 on *B. subtilis* and *E. coli* ribosomes, whereas ArfA and *E. coli* RF2 only act on *E. coli* non-stop complexes. Additionally, RF2 is not interchangeable between ArfA and BrfA, indicating that the systems are adapted to the corresponding species. The low interspecies compatibility is in accordance with the late origin and hence narrow phylogenetic distribution of these alternative release systems. Although BrfA and ArfA share a regulatory mechanism and are specific for RF2 of their respective species, the different phylogenetic distribution and the distinct amino acid composition hint toward an unrelated evolution of the systems (Shimokawa-Chiba et al., 2019). The recent cryo-EM structure of *B. subtilis* BrfA and *B. subtilis* RF2 on the *E. coli* non-stop ribosomal complex shed light into the mechanism of ribosome rescue by the BrfA/RF2 system (Shimokawa-Chiba et al., 2019). Comparison of BrfA and ArfA structures shows that the interactions formed by the N-termini are very distinct. While the N-terminus of ArfA loops back toward the decoding center, the BrfA N-terminus forms an α-helix, which binds between the large and the small subunit (Figures 3F,G). Afterward, BrfA proceeds toward the decoding center and extends into the mRNA channel. The closed conformation was not observed in the BrfA dataset, hence BrfA efficiently activated the open conformation of RF2. The overall conformation of RF2 is similar in the structure of the BrfA/RF2 and the ArfA/RF2 system, in both the catalytic domain III of RF2 is directed toward the PTC (James et al., 2016; Demo et al., 2017; Huter et al., 2017c; Ma et al., 2017; Zeng et al., 2017; Shimokawa-Chiba et al., 2019; Figures 3F,G). As observed for ArfA, recruitment of RF2 by BrfA is mediated *via* addition of a β-strand to the β-sheet of the superdomain II/IV, where the β-strand of the rescue factor lies between RF2 and the decoding center (James et al., 2016; Demo et al., 2017; Huter et al., 2017c; Ma et al., 2017; Zeng et al., 2017; Shimokawa-Chiba et al., 2019). BrfA binds to the mRNA entry channel *via* positively charged residues in the C-terminal region (Shimokawa-Chiba et al., 2019), however, mutational analysis of the BrfA C-terminus is not available yet. Remarkably, the C-termini of ArfA and BrfA both have a lysine-histidine (KH) motif which binds to a pocket in the mRNA entry channel (James et al., 2016; Demo et al., 2017; Huter et al., 2017c; Ma et al., 2017; Zeng et al., 2017; Shimokawa-Chiba et al., 2019). This motif is the only outstanding sequence similarity between ArfA and BrfA and if these amino acids are important for both factors could be the subject of future experiments.

### Release Factor Independent Alternative Ribosome Rescue Factors

**Alternative ribosome rescue factor B (ArfB, formerly YaeJ)** is currently the only known Arf which is RF independent. It was identified as a multicopy suppressor upon deletion of *trans*-translation and ArfA in *E. coli*, and was additionally shown to hydrolyze peptidyl-tRNA on non-stop complexes *in vivo* and *in vitro* by itself (Chadani et al., 2011b; Handa et al., 2011). Deletion of *trans*-translation and ArfA is synthetically lethal in *E. coli* despite a chromosomal *arfB* gene, hence the physiological role of ArfB is not clear yet. By contrast, in *Caulobacter crescentus*, ArfB of chromosomal origin ensures survival and is essential in the absence of *trans*-translation. Also, most eukaryotes have an ArfB homolog that is targeted to mitochondria, whereas ArfA is not found in eukaryotes and *trans*-translation is only conserved in mitochondria of some protists (Duarte et al., 2012). Best characterized is the homolog of human mitochondria, named immature colon carcinoma transcript-1 (ICT1), which is essential for cell viability (Handa et al., 2010; Richter et al., 2010; Feaga et al., 2016). Although ICT1 was identified to be integrated into the large subunit of the mitochondrial ribosome (mitochondrial large subunit protein 58, MRPL58, also mL62), it rescues *E. coli* and mammalian mitochondrial non-stop complexes *in vitro* (Richter et al., 2010; Koc et al., 2013; Akabane et al., 2014; Brown et al., 2014; Greber et al., 2014a, b; Kogure et al., 2014; Feaga et al., 2016). ArfB and ICT1 are functionally interchangeable *in vivo*, since ICT1 can complement the synthetic lethal phenotype of double deletion of ArfB and *trans*-translation in *C. crescentus*, and plasmid derived ArfB supports viability of human cells upon ICT1 knock-down (Feaga et al., 2016). However, whether ICT1 or another putative mitochondrial peptidyl-hydrolase releases non-stop complexes in mitochondria remains contentious (Richter et al., 2010; Duarte et al., 2012; Akabane et al., 2014; Chrzanowska-Lightowlers and Lightowlers, 2015; Takeuchi and Nierhaus, 2015; Ayyub et al., 2020).

Alternative ribosome rescue factor B consists of 140 amino acids, and has a NTD (residues 1–100) and a C-terminal tail (residues 115–140), connected *via* a ∼12 amino acid long flexible linker (Gagnon et al., 2012; Chan et al., 2020). The NTD is homologous to domain III of bacterial class I RFs, including the GGQ motif, whereas further class I RF domains, like the codon recognition superdomain II/IV, are absent (Singarapu et al., 2008; Chadani et al., 2011b; Gagnon et al., 2012; Kogure et al., 2014; Chan et al., 2020). As for class I RFs mutation of the GGQ (G_25_G_26_Q_27_) motif abolished peptidyl-tRNA hydrolysis activity (Korostelev et al., 2008; Chadani et al., 2011b; Handa et al., 2011; Santos et al., 2013). The C-terminal tail has some similarities to the C-terminal tail of SmpB, as it contains positively charged amino acids, stays unstructured in solution and engages an α-helical conformation upon binding to the ribosome (Chadani et al., 2011b; Gagnon et al., 2012; Kogure et al., 2014; Chan et al., 2020). Sucrose density gradient centrifugation showed that ArfB is associated with 70S ribosomes, as well as polysomes (Chadani et al., 2011b; Handa et al., 2011), and that removing 10 C-terminal residues abolished binding to the ribosome (Handa et al., 2011). Further mutational analysis showed that mutating three positively charged residues to alanine already severely decreased binding affinity for the ribosome (Kogure et al., 2014). By contrast, the single mutations in the C-terminal tail or removal of two amino acids from the linker did not influence binding to the ribosome, although hydrolysis activity was lost as a consequence.

**FIGURE 4:**
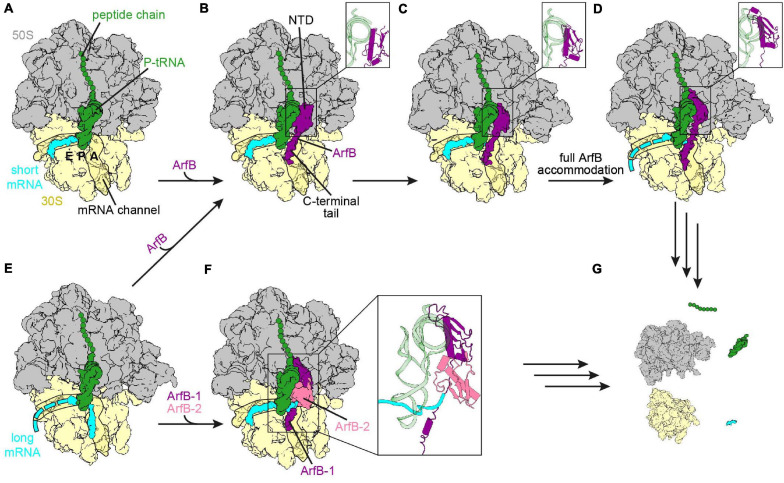
Ribosome rescue by ArfB. **(A)** Non-stop complex (large subunit, 50S, gray; small subunit, 30S, yellow) with peptidyl-tRNA (green) in the P-site and mRNA extending two nucleotides into the A-site (short mRNA, cyan) [PDB ID 7JSZ (Carbone et al., 2020)]. **(B-D)** ArfB (purple) bound to the non-stop complex with the NTD in the collapsed **(B)** [PDB ID 7JSZ (Carbone et al., 2020)], pre-accommodated **(C)** [PDB ID 7JSW (Carbone et al., 2020)], as well as fully accommodated **(D)** [PDB ID 6YSS (Chan et al., 2020)] conformation. The conformation of the NTD is enlarged in the inlays. The C-terminal tail extends into the mRNA channel in each conformation. **(E)** Ribosome with mRNA extending nine nucleotides into the A-site and the mRNA channel (long mRNA, cyan) and peptidyl-tRNA in the P-site [PDB ID 6YSR (Chan et al., 2020)]. **(F)** ArfB dimer (ArfB, purple and ArfB-2, pale violet red) on the ribosome with the long mRNA sandwiched between the ArfB dimer and the P-site tRNA [PDB ID 7JT1 (Carbone et al., 2020)]. **(G)** ArfB or the ArfB dimer dissociated and ribosome recycling had occurred.

A recent cryo-EM study revealed two intermediate steps of ArfB accommodation to a non-stop complex (Carbone et al., 2020). In the first intermediate the C-terminal tail has already bound to the mRNA entry channel and formed an α-helix, while the NTD is associated with H69 of the 23S rRNA and the flexible GGQ loop is pointed away from the A-site of the 50S subunit (Figures 4A,B). The second intermediate shows that the NTD is still bound to H69, but is rotated so that the GGQ loop is directed toward the large subunit A-site (Figure 4C), priming ArfB for insertion into the PTC to perform hydrolysis. Eventually, the NTD is placed in the A-site of the large subunit and accommodates into the PTC (Figure 4D), which is induced upon binding of the NTD (Gagnon et al., 2012; Carbone et al., 2020; Chan et al., 2020). The GGQ motif adopts an analogous conformation to the GGQ motif of bacterial class I RFs and mediates hydrolysis of the ester bond between the peptide and the P-tRNA. During all stages the position of the tail overlaps with the path of a full-length mRNA, as well as with C-terminal tail of SmpB and the C-terminus of ArfA, indicating that all of these systems monitor the channel (Gagnon et al., 2012; Neubauer et al., 2012; James et al., 2016; Demo et al., 2017; Huter et al., 2017c; Zeng et al., 2017; Chan et al., 2020). As for *trans*-translation, the release activity decreased with increasing length of the mRNA, with a significant drop for mRNAs with more than nine nucleotides downstream of the P-site (Ivanova et al., 2004; Feaga et al., 2016). By contrast, ArfB and the tmRNA⋅SmpB complex were shown to associate with the ribosome independently of the mRNA length (Kurita et al., 2014b; Chan et al., 2020). This indicates that the mRNA and the C-terminal tails of ArfB and SmpB can compete with the mRNA for binding to the mRNA channel. In case of longer mRNAs dissociation of ArfB and tmRNA⋅SmpB is preferred over binding to the mRNA, so that an interference during the translation cycle is unlikely (Kurita et al., 2014b; Chan et al., 2020). Why ArfA has a stricter mRNA length dependency (Ivanova et al., 2004; Asano et al., 2005; Shimizu, 2012; Kurita et al., 2014b; Zeng and Jin, 2016), and thus competes less with the mRNA about binding to the mRNA channel remains to be determined.

Upon ArfB binding, the decoding center adopts a specific conformation (Gagnon et al., 2012; Chan et al., 2020), such that the 16S rRNA nucleotide G530 is in *anti*-conformation, but the positioning differs from the ON conformation seen with an A-tRNA (Gagnon et al., 2012; Huter et al., 2017b). A1492 of the 16S rRNA is located within h44, while A1493 is rotated out of h44 and stacks upon A1913 of H69 of the 23S rRNA. Proline 110 of ArfB stacks onto A1493 and was proposed to act as a “hinge,” restricting the movement of the tail on one side, while allowing movement and accommodation of the NTD on the other (Gagnon et al., 2012). However, this proline is not conserved throughout ArfB proteins, and can be mutated to alanine without loss of activity. Instead, the nearby Arg_105_ is important for the activity of ArfB, since its mutation to alanine reduced the activity by 80% (Kogure et al., 2014). Indeed, the sidechain stacks on U1915 of H69, while C1914, which usually stacks on U1915 [e.g. during termination (Korostelev et al., 2008; Laurberg et al., 2008; Weixlbaumer et al., 2008; Korostelev et al., 2010), and in the presence of A-site tRNA (Arenz et al., 2016), ArfA/RF2 (James et al., 2016; Demo et al., 2017; Huter et al., 2017c; Ma et al., 2017; Zeng et al., 2017), or SmpB⋅tmRNA (Rae et al., 2019)], respectively flips out of H69 (Gagnon et al., 2012; Chan et al., 2020). Interestingly, Korostelev and co-workers formed a non-stop complex with two mRNA nucleotides reaching into the A-site (Carbone et al., 2020). In this complex the decoding center adopts a different conformation in which A1492 and A1493 are flipped into h44 and stack with the overhanging mRNA nucleotides. Since A1493 is not rotated out of h44, Pro110 of ArfB cannot stack with it, so that the interactions of the linker are changed. Furthermore, Arg_105_ does not interact with U1915 although the NTD is accommodated to the PTC. This conformational flexibility of the decoding center and the ArfB linker region demonstrate the ability of both to adapt to different mRNA lengths. The dissociation mechanism of ArfB is still unknown, but it has been reported recently that the dissociation of ArfB is rather slow and could be facilitated by rotation of the ribosome subunits following hydrolysis of the peptide chain (Carbone et al., 2020; Chan et al., 2020).

Recently cryo-EM structures of ArfB bound to ribosomes with a mRNA extending nine nucleotides past the P-site were published (Carbone et al., 2020; Chan et al., 2020). In both structures the mRNA had moved out of the mRNA entry channel. However, while Chan et al. (2020) observed that the mRNA was flexible after the P-site and ArfB was accommodated in the same manner as with mRNAs truncated after the P-site (Figures 4B–E), Carbone et al. (2020) found that the mRNA overhang was sandwiched by the P-site tRNA and an ArfB dimer (Figure 4F). In the dimer, one ArfB was bound to the ribosome in the same way as seen in the presence of non-stop mRNAs, with the NTD reaching into the PTC and the C-terminal tail forming an α-helix in the mRNA channel. The binding site of the NTD of the second ArfB (ArfB-2) was flipped compared to the first ArfB, i.e., with the flexible GGQ loop directed toward the small subunit instead of the PTC on the large subunit, while the C-terminal tail was unstructured and not resolved. Dimerization was mediated by the N-terminal amino acids, which formed an antiparallel β-sheet. Whether both pathways co-exist in the bacterial cell or one is preferred is not known yet and could be the subject of future experiments. The current hypothesis for the dissociation of the dimer is similar to the one proposed for ArfB dissociation from the non-stop complex with a short mRNA. The dimer dissociation was suggested to be mediated by subunit rotation with ArfB-2 being proposed to leave the ribosome first (Carbone et al., 2020). Regardless of whether a monomer or dimer of ArfB participates in the rescue, the ribosome is recycled eventually (Figure 4G) allowing the subunits to be reused for subsequent rounds of translation (Gagnon et al., 2012; Chan et al., 2020).

### Bacterial Ribosome-Associated Quality Control

Recently, bacterial RQC involving C-terminal non-templated (i.e., mRNA independent) tailing of the nascent polypeptide chain, analogous to eukaryotic Rqc2-mediated RQC, was described in *B. subtilis* (Lytvynenko et al., 2019). For this process to occur, the ribosomal subunits must separate during translation elongation without release of the nascent polypeptide, resulting in a large ribosomal subunit in complex with peptidyl tRNA-nascent chain. In eukaryotes, ribosomal subunits can be dissociated by Hbs1/HBS1L and Dom34/Pelota (additionally requiring ABCE1 in mammals) in the case of non-stop translation (Shoemaker et al., 2010; Pisareva et al., 2011). Alternatively, ribosome ubiquitylation, for example of collided ribosomes, can trigger subunit dissociation, which requires the RQT complex (Matsuo et al., 2017; Sitron et al., 2017). By contrast, the mechanism of subunit splitting prior to RQC in bacteria is currently unknown. Induction of splitting and the following RQC could be a response to stresses such as heat, antibiotics or translational stalling (Lytvynenko et al., 2019). The NEMF-family protein RqcH, which is widely distributed but has been lost multiple times during bacterial evolution, binds to the obstructed large subunit and extends the nascent polypeptide chain with C-terminal alanine tails (Burroughs and Aravind, 2014; Lytvynenko et al., 2019). Yeast Rqc2p adds C-terminal tails consisting of both alanine and threonine (Shen et al., 2015), while it has recently been shown that in mammals, like in bacteria, the tails consist mostly of alanine (Udagawa et al., 2021). In eukaryotes, the process is terminated by Vms1/ANKZF1, perhaps facilitated by the ABCF protein Arb1 (Kuroha et al., 2018; Verma et al., 2018; Su et al., 2019). How the tailing process is terminated in bacteria remains unknown. After extraction of the polypeptide chain, the C-terminal alanine tail serves as a degradation signal, which is recognized by the protease ClpXP (Lytvynenko et al., 2019).

**FIGURE 5:**
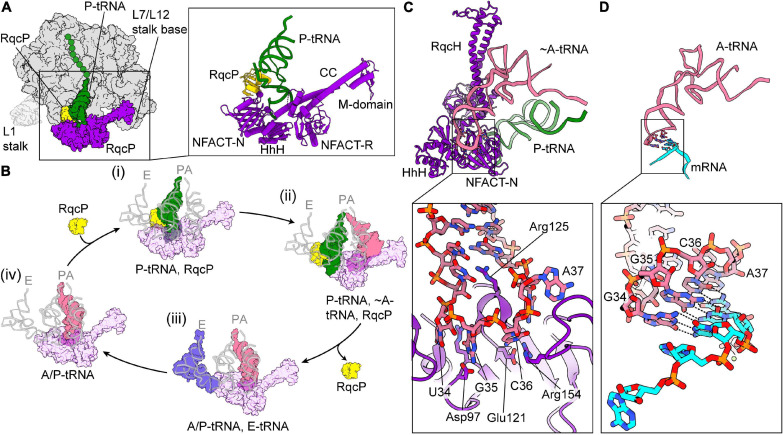
Ribosome-associated quality control mediated by RqcH in *Bacillus subtilis*. **(A)** Overview of the large subunit (50S, gray) with P-tRNA–nascent chain (green), RqcH (purple), and RqcP (yellow) bound [PDB ID 7AS8 (Crowe-McAuliffe et al., 2021)]. The approximate position of the L1 stalk, which was disordered in this structure, is shown faintly. On the right, a close-up of RqcH, RqcP, and P-tRNA is shown with the domains of RqcH indicated. **(B)** Proposed mechanism for C-terminal alanine tailing mediated by RqcH and RqcP. For comparison, positions of canonical A-, P-, and E-tRNAs are overlaid in faint gray [PDB ID 6CFJ (Tereshchenkov et al., 2018)]. The large subunit and nascent chain have been omitted for clarity. (i) RqcH (transparent purple) and RqcP (yellow) bound to P-tRNA–nascent chain (green), as in **(A)**. RqcH may also dissociate from this state. (ii) The pre-peptidyl transfer state. The incoming tRNA is positioned similarly to an A-tRNA (salmon) [PDB ID 7AQC (Filbeck et al., 2021)]. (iii) Subsequent to peptidyl transfer and RqcP dissociation, the P-tRNA has moved to the E-site (blue) [State C described in Crowe-McAuliffe et al., 2021). (iv) Similar to (iii), except the E-tRNA has dissociated. Binding of RqcP and movement of RqcH and the L7/L12 stalk completes the cycle [PDB ID 7AS9 (Crowe-McAuliffe et al., 2021)]. **(C)** The RqcH NFACT-N and HhH domain “decode” tRNA^*Ala(UGC)*^ on the 50S, same as state **(B)** (ii) [PDB ID 7AQC (Filbeck et al., 2021)]. The tRNA anticodon is splayed in a distorted conformation. **(D)** Same view as **(C)**, showing conventional interaction of tRNA^*Ala(GGC)*^ with mRNA on a 70S ribosome [PDB ID 6OF6 (Nguyen et al., 2020)] **(C,D)** were aligned by the tRNA.

RqcH is a homolog of the eukaryotic RQC factor Rqc2/NEMF (recently reviewed by Joazeiro, 2019 and Yan and Zaher, 2019). These factors have a conserved core domain architecture of NFACT-N (NFACT means domain found in NEMF, FbpA, Caliban, and Tae2), helix-hairpin-helix (HhH), coiled coils (CC), a globular middle (M)-domain between the two helices of the CC, and an NFACT-R domain (Burroughs and Aravind, 2014; Shao et al., 2015; Shen et al., 2015; Lytvynenko et al., 2019; Crowe-McAuliffe et al., 2021). Eukaryotic NEMF proteins typically contain an additional NFACT-C domain of unknown function (Burroughs and Aravind, 2014). Recently, two groups have analyzed bacterial RQC by cryo-EM, using samples prepared by immunoprecipitation of a FLAG-tagged RqcH in *B. subtilis* (Crowe-McAuliffe et al., 2021; Filbeck et al., 2021; highlighted by Brandman and Frost, 2021). In both structures, RqcH was bound to the large subunit–P-tRNA–nascent chain complex, straddling the central protuberance (CP) and the L7/L12 stalk, similarly to what was observed in low-resolution reconstructions of eukaryotic NEMF proteins (Shao et al., 2015; Shen et al., 2015). The NFACT-N and HhH domains interacted with peptidyl-tRNA, while the CC domain spanned the translation-factor binding site, allowing the M domain to interact with the L7/L12 stalk base (Figure 5A). The NFACT-R domain, which was relatively poorly resolved, was positioned close to the A-site finger. In both studies, extensive *in silico* classification was required to resolve structures, implying highly heterogeneous samples. One class, which contained RqcH and an A/P-like peptidyl tRNA, was highly similar between both datasets. Both groups also observed an additional protein, RqcP (ribosome quality control P-tRNA, formerly YabO), in classes that contained a peptidyl-tRNA bound at the P-site on the large subunit. RqcP was seen to bind between H69 of the 23S rRNA, the anticodon stem of the P-tRNA and the NFACT-N domain of RqcH (Figure 5A). RqcP co-precipitates RqcH, and is required for alanine tailing (Crowe-McAuliffe et al., 2021; Filbeck et al., 2021).

RqcP belongs to the widely distributed S4 RNA-binding family, and is homologous to *E. coli* heat shock protein 15 (Hsp15). Hsp15 is upregulated after heat shock and has previously been implicated in an RQC-like process, as it associates with obstructed 50S subunits *in vitro* (Korber et al., 2000; Jiang et al., 2009). However, several lines of evidence indicate that Hsp15 and RqcP are functionally distinct:

1. *E. coli* does not have an RqcH homolog, and hence Hsp15 cannot function in RQC.
2. Hsp15 has a conserved C-terminal extension, which, remarkably, is conserved only in the polyphyletic species not harboring an *rqcH* gene (Crowe-McAuliffe et al., 2021).
3. Unlike Hsp15, RqcP is not upregulated upon heat shock (Nicolas et al., 2012).
4. *E. coli* Hsp15 cannot functionally substitute for RqcP in *B. subtilis* (Crowe-McAuliffe et al., 2021).

A recent analysis of RQC in mitochondria also identified an S4-domain-containing protein apparently acting analogously to RqcP in *B. subtilis* RQC complexes (Desai et al., 2020). In this study, ribosomes from mitochondria deficient for an elongator tRNA, a condition predicted to cause translational stalling, were isolated. From a large number of initial micrographs, a population of particles consisting of large subunit–P-tRNA–nascent chain complexes were identified by *in silico* classification. A protein containing an S4 domain, MTRES1, was observed binding in a similar position to RqcP, and interacting with a non-canonical RF homolog, mtRF-R (previously C12orf65) (Desai et al., 2020).

Based on their cryo-EM data and extensive *in silico* classification, Crowe-McAuliffe et al. (2021) postulated a mechanism for alanine tailing of the nascent polypeptide chain by RqcH and RqcP (Figure 5B): After splitting by an unknown process, the peptidyl-tRNA bound to the large subunit is bound by RqcP, which is positioned close to H69 of the large subunit and contacts the ASL of the peptidyl-tRNA. This interaction places the peptidyl-tRNA in the P-site and primes the obstructed large subunit for the first round of alanine tailing, and is compatible with RqcH binding in subsequent rounds of elongation (Figure 5B i). Next, RqcH and alanine-tRNA^*Ala*^ bind to the obstructed large subunit, which could happen either successively or in the form of a complex. The following step was hypothesized to contain a large subunit with P-tRNA–nascent chain, RqcP, RqcH, and an Ala-tRNA^*Ala*^ in the A-site (Figure 5B ii). However, this state was predicted to be labile and was not observed, perhaps due to rapid peptidyl transfer. The RqcH NFACT-N domain interacts with the ASL of alanine-tRNA^*Ala*^ in the A site, thereby poising the complex for peptidyl transfer, which results in a deacylated tRNA in the P-site and peptidyl-tRNA^*Ala*^ in the A-site. The P-site tRNA is then transferred to the E-site before dissociating, which requires RqcP to leave and “clear the path” between the P- and E-sites (Figure 5B iii). This is concomitant with a scissor-like movement of RqcH, in which the coils of the CC domain shift in conformation. After dissociation of the E-tRNA, the peptidyl-tRNA^*Ala*^ is then positioned between the A- and P-site (A/P-state), while the ASL is still bound to RqcH (Figure 5B iv). Association of RqcP with this complex is coincident with a translocation-like event as the peptidyl-tRNA^*Ala*^ enters the P-site, additionally RqcH and the L7/L12 stalk also shift. The A-site is then free for the next round of alanine-tailing. It is not clear whether a new alanine-tRNA^*Ala*^ is recruited to the complex or if RqcH dissociates and reassociates with alanine-tRNA^*Ala*^ to the obstructed large subunit. The alanine tails are extended and the tailed polypeptide is extracted from the large subunit after several rounds of alanine tailing in an unknown mechanism. *In toto*, the heterogenous states observed in this sample resembled tRNA states that occur during regular translation. Although the presence of additional steps cannot be excluded, this scheme notably does not involve any conventional translation factors or GTPases, consistent with *in vitro* analysis of CAT tailing in eukaryotes (Osuna et al., 2017).

The study from Filbeck et al. (2021), placed focus on one class containing a large subunit with P-tRNA–nascent chain, RqcP, RqcH, and an A-tRNA—a state hypothesized to exist but not directly observed in the other study (Crowe-McAuliffe et al., 2021; Figure 5B ii). The reason for this discrepancy is unclear, although it may be due to addition of methanol or additional tRNA to the immunoprecipitated samples. Close inspection of the cryo-EM map reveals that density for the A-site tRNA terminates at the CCA-3′ end, indicating that this tRNA is not aminoacylated and explaining why peptidyl transfer could not occur. The relatively high resolution of the reconstruction allowed a detailed analysis of how RqcH interacts with the tRNA^*Ala*^ anticodon, identifying a network of interactions between the RqcH NFACT-N domain and the tRNA anticodon, which is dramatically splayed out (Figure 5C) compared to a regular decoding conformation (Figure 5D). This is consistent with proposed action of Rqc2p in yeast (Shen et al., 2015), as well as the model from Crowe-McAuliffe et al. (2021).

Several questions about bacterial RQC remain unanswered. What conditions trigger subunit dissociation, and does a dedicated factor mediate this process? Is there an analog of RqcP in eukaryotic RQC? How does RqcH recognize tRNA^*Ala*^ when not bound to the ribosome? How is the tailing process terminated?

## Conclusion and Outlook

The structural and biochemical data obtained over the last decade has shed much-needed light onto the diverse ribosome rescue mechanisms present in bacteria. Despite the apparent similarities between ArfA and BrfA involving regulation of expression by *trans-*translation, recruitment of RF2 and the KH motif, the very low sequence similarity indicates that the systems evolved independently (Shimokawa-Chiba et al., 2019). This also holds true for *F. tularensis* ArfT, which is only ∼40 amino acids in total (Goralski et al., 2018; Shimokawa-Chiba et al., 2019). In this regard further yet-unidentified rescue mechanisms involving small proteins that can recruit and activate RFs could exist in other bacterial species. Identification of those could be rather difficult because the systems could possibly not be identified by sequence similarities and need genetic screening in *trans-*translation deleted backgrounds. Interestingly, Lytvynenko et al. (2019) found that RQC occurs *in vivo* as a response to stalling on a non-stop reporter gene when *trans*-translation is inactivated by *ssrA* deletion. Double deletion of the *ssrA* and the *rqcH* genes was not synthetically lethal under normal growth conditions, perhaps because of the presence of BrfA (Shimokawa-Chiba et al., 2019). However, growth was strongly inhibited upon heat or antibiotic stress. The interplay between *trans-*translation, BrfA and RqcH has not been investigated yet, but apparently BrfA was not able to suppress the growth defect upon stress (Lytvynenko et al., 2019). *Vice versa* RqcH was not able to overcome the synthetic lethality upon double deletion of *trans-*translation and the *brfA* gene (Shimokawa-Chiba et al., 2019). However, for the synthetic lethality screen the *brfA* gene was replaced by an antibiotic resistance gene, conferring resistance to erythromycin, and the use of the antibiotic during the screen might have stressed the cells, so that it could be possible that without an antibiotic deletion of *trans-*translation, BrfA and RqcH is necessary to induce synthetic lethality in *B. subtilis*. This is supported by knockdowns by CRISPR interference, which show synthetic lethality upon cold stress, but not at 37°C, where only a strong growth defect was observed (Shimokawa-Chiba et al., 2019). However, the three mechanisms seem to have at least partially overlapping cellular responsibilities and to further elucidate the interplay between them additional experiments are needed.

The activity of the BrfA/RF2 and the *trans*-translation system has also been investigated with ribosomes stalled by the *B. subtilis* MifM arrest sequence, which specifically stalls *B. subtilis* ribosomes (Sohmen et al., 2015; Shimokawa-Chiba et al., 2019). Arrest sequences usually serve in the regulation of a downstream ORF and mediate stalling during their own translation by interaction with the nascent polypeptide exit tunnel (NPET) (reviewed by Ito and Chiba, 2013; Arenz et al., 2014; Wilson et al., 2016). In particular, arrest sequences interactions with the NPET occur from the PTC to the constriction formed by the ribosomal proteins L4 and L22, which influences the activity of the PTC probably *via* an allosteric relay. As reported for ArfA/RF2, ArfB and *trans*-translation with respect to *E. coli* ribosomes stalled by the *E. coli* SecM arrest sequence (Garza-Sánchez et al., 2006; Chadani et al., 2012), the observed activity of BrfA/RF2 and *trans*-translation of MifM-stalled *B. subtilis* ribosomes was low and not above the background level of spontaneous peptidyl-tRNA hydrolysis (Shimokawa-Chiba et al., 2019). However, during stalling on arrest peptides, the mRNA entry channel is usually occupied and the mRNA would be quite long, since another ORF follows downstream of the channel, both rendering arrest peptide stalled ribosomes a poor target for ribosome rescue mechanisms (Ivanova et al., 2004; Shimizu, 2012; Kurita et al., 2014a; Feaga et al., 2016; Zeng and Jin, 2016; Shimokawa-Chiba et al., 2019; Chan et al., 2020). By contrast, the inhibition of *trans*-translation with respect to SecM-stalled ribosomes was mainly due to binding of proline-tRNA^*Pro*^ to the ribosomal A-site, which prevented binding of tmRNA⋅SmpB (Garza-Sánchez et al., 2006). In the absence of proline-tRNA^*Pro*^ *trans*-translation was less inhibited by SecM stalling. Furthermore, mRNA cleavage in the A-site due to prolonged stalling was inhibited by the presence of proline-tRNA^*Pro*^ (Garza-Sánchez et al., 2006). Nevertheless, degradation of the mRNA would be possible until the nucleases encounter the entry of the mRNA entry channel, which protects the mRNA from further cleavage (Sunohara et al., 2004; Garza-Sánchez et al., 2006). The remaining mRNA would extend approximately 12–15 nucleotides past the P-site and represent an mRNA overhang upon which *trans*-translation can still act (Ivanova et al., 2004). This explains the activity of *trans*-translation on SecM-stalled ribosomes in the absence of proline-tRNA^*Pro*^. The same principle could also apply to MifM arrest, because stalling occurs on several sequential codons due to slow peptide bond formation with a sense codon in the A-site (Chiba and Ito, 2012), which allows binding of the cognate tRNA. Interestingly, biochemical experiments showed that introduction of a stop codon into the MifM arrest sequence did not lead to termination (Chiba and Ito, 2012). This was also observed for a further arrest peptide, called TnaC, which allowed peptide bond formation but specifically inhibited termination (Gong and Yanofsky, 2002). Superimposition of the structures of TnaC and MifM stalled ribosomes show a similar conformation for the 23S rRNA nucleotide A2602 (Seidelt et al., 2009; Bischoff et al., 2014; Sohmen et al., 2015), which usually supports binding of the GGQ motif in domain III of RFs to the PTC (Korostelev et al., 2008, 2010; Laurberg et al., 2008; Weixlbaumer et al., 2008). However, during stalling the conformation of A2602 is restricted and the induced state of the PTC upon RF binding is inhibited, hence accommodation of the GGQ motif and subsequent peptidyl-tRNA hydrolysis are inhibited as well (Seidelt et al., 2009; Bischoff et al., 2014; Sohmen et al., 2015). This could indicate that after occasional BrfA/RF2 binding and activation during MifM stalling, the adopted conformation A2602 may further inhibit BrfA/RF2 action by preventing accommodation of the GGQ motif at the PTC. Whether specific inhibition of termination also occurs with *E. coli* SecM, *E. coli* ErmBL, *S. aureus* ErmCL or VemP from *Vibrio alginolyticus* is not known yet, but superimposition of ribosome bound, open RF2 with the respective structure of the ribosome with SecM, ErmBL, ErmCL, or VemP bound in the NPET also indicates conformations of A2602 that are incompatible with accommodation of the GGQ motif (Arenz et al., 2014, 2016; Zhang et al., 2015; Su et al., 2017).

Bacterial ribosome rescue mechanisms are a promising target for development of novel antibiotics, especially since most such systems appear to have no direct homolog in mammals (i.e., *trans-*translation, ArfA, BrfA, and ArfT). It has been shown that a synthetic peptide, which is equivalent to the C-terminal tail of SmpB inhibits binding of tmRNA⋅SmpB to the A-site of the ribosome, as well as peptidyl transfer to the TLD of tmRNA (Kurita et al., 2010; Mace et al., 2017). Furthermore, *trans-*translation is conserved in all sequenced bacterial genomes (Keiler and Feaga, 2014), thus the peptide might affect a broad range of bacteria. It will be interesting to see if similar strategies can be applied with synthetic peptides corresponding to the C-termini of ArfA, ArfB, or BrfA. The broad distribution of ArfB also renders its C-terminal tail an interesting candidate for such investigations (Burroughs and Aravind, 2019). Furthermore, the KH motif, which is conserved between ArfA and BrfA displays conserved interactions with the mRNA entry channel (James et al., 2016; Demo et al., 2017; Huter et al., 2017c; Ma et al., 2017; Zeng et al., 2017; Shimokawa-Chiba et al., 2019), which could indicate a binding of the C-termini of both systems to ribosomes of different species. Also, specific inhibition of *trans-*translation for species, in which *trans-*translation is essential or in combination with other antimicrobial agents would be possible by the usage of an antisense DNA oligonucleotide to the gene encoding tmRNA (*ssrA* gene). This approach was already applied *in vitro* using an antisense oligonucleotide to the MLD of tmRNA, which blocked the activity of *trans-*translation (Hanes and Pluckthun, 1997). Furthermore, peptide aptamers were developed against *trans-*translation and ArfA of *Aeromonas veronii* and the expression of an aptamer against one of the systems from a plasmid led to reduced growth (Liu et al., 2016). In 2013, members of a class of small molecules, the so-called KKLs, showed broad-spectrum antibiotic activity and were proposed to inhibit *trans*-translation *in vitro* and *in vivo* (Ramadoss et al., 2013). However, it was challenged that *trans*-translation is the only or even major target of KKLs (Mace et al., 2017; Brunel et al., 2018; Tresse et al., 2019) and the subject is matter of an ongoing discussion (Aron et al., 2020; Guyomar et al., 2020). This has not however deterred attempts to develop more sensitive and selective high-throughput screening assays (Guyomar et al., 2020; Thépaut et al., 2020) but only time will tell whether they will lead to discovery of the elusive molecules that specifically target and inhibit bacterial ribosome rescue systems.

## Author Contributions

CM and CC-M made the figures. CM wrote the manuscript with help from CC-M and DW. All authors contributed to the article and approved the submitted version.

## Conflict of Interest

The authors declare that the research was conducted in the absence of any commercial or financial relationships that could be construed as a potential conflict of interest.
